# On the Author of the Theory Concerning the Secretion of Pus

**Published:** 1817-09

**Authors:** James Curry


### *For the London Medical and Physical Journal.*

### *On the Author of the Theory concerning the Secretion of Pus;* by JAMES CURRY, M.D.

FOR your being the first in communicating to the British faculty the ingenious and candid critique, by the New-England Journalists, upon Caldwell's Notes, &c. on Cullen's First Lines, accept my grateful acknowledgment. I equally approve of your candour respecting the disputed claim to the Glandular theory of Suppuration, and trust that the opinion which *I* have taught in the medical school of Guy's Hospital for sixteen years past, will, when explained, not be found to call for more than the "regret" you express "at hearing of such doctrines being promulgated in one of the London schools" (Med. and Phys. Journal, No. 222 for July 1817, note *, on p. 163), viz. that Dr. Morgan of Philadelphia, and not Mr. John Hunter, was the first who affirmed that *pus was a secretion.* Now, sir, I am proud thus publicly to avow, that, in the doctrines I have taught, and, God willing, shall ever teach, I have never been guided by any other motive than a sincere belief of their being well founded; and that, although not devoid of national partiality, where that is justifiable, I have never yet sacrificed truth to national attachment. On this ground alone I have always followed the precept of *detur digniori*, and awarded praise where praise was justly due, without a moment's consideration of the claimant's æra, or country: "*Tros Tyriusve mihi nullo discrimine agetur.*" Although, perhaps, *in a practical view*, the question respecting the formation of pus is of little consequence, yet I have always been impressed with a conviction, that what many hold to be mere speculative questions in science, are, nevertheless, worthy of notice, upon this principle, that, in natural knowledge, every fact has a bearing, however remote, upon some other and more important ones, which, perhaps, yet remain to be discovered, but which *this seemingly insulated one* will serve to illustrate and confirm. Thinking so, I could not pass over unnoticed a point which had been successively laboured on by Boerhaave, Grashius, De Haen, Quesnay, Pringle, Morgan, and John Hunter;

Hunter; and, allowing any merit to the *glandular theory*, I could not avoid giving that merit to Dr. Morgan, who discussed the question with great ingenuity in his Inaugural Dissertation, on taking his degree at Edinburgh in 1763; whilst I could find no proof that Mr. Hunter had taught, or even adopted, such opinion, until a considerably later period: for Sir Everard Home, who, in 1788, published a "Dissertation on the Properties of Pus," and claims the merit of the doctrine as entirely belonging to Mr. Hunter, has altogether forgotten to support his claim, by stating at what time it was first formed and promulgated by him. It is, indeed, very possible, that Sir Everard did not think such a step at all necessary; for he never once notices Dr. Morgan's Thesis, and might not even know that any such thing existed. It is often said, and, in certain things, I think it true, that we grow fond of early opinions, and more tenacious of them as we become older; but I shall feel sincere gratification in being set right, if I am wrong, upon this point, and shall not fail to make the *amende honorable* to the memory of John Hunter, by transferring from Dr. Morgan to our British Machaon the exclusive claim to originality. I may here remark, by the way, that the first *hint* of this doctrine will be found in Dr. Simson's "Tractatus de Re Medica," published in 1740, in which he asks, "may not an issue be considered as performing the office of a gland?" and I think it not improbable but that Dr. Morgan took this *hint*, as Simson's book, though now little valued or read, was then common in the hands of students at Edinburgh.

After having said so much upon this question, it may seem odd that I should wind up by stating that I believe the doctrine itself *untrue*, nay more, that it is even completely overturned by the very experiments which have been adduced in its favour. The detail of arguments, however, to prove what I here advance, would take up much more time than I can at present allot to it; but, should the question be thought worthy of farther discussion, I pledge myself to make good the conclusion I have drawn.

*Bridge-street, Blackfriars;*
*August* 1, 1817.

---

**We shall feel extremely thankful to Dr. Curry by the honour of a further communication on this interesting subject; and we trust Mr. Hunter's friends will not be backward in defending their master.—EDIT.**

COLLECTANEA.

